# Taking on *Plasmodium vivax* malaria: A timely and important challenge

**DOI:** 10.1371/journal.pmed.1003593

**Published:** 2021-04-23

**Authors:** Lorenz von Seidlein, Nicholas J. White

**Affiliations:** 1 Mahidol Oxford Tropical Medicine Research Unit, Faculty of Tropical Medicine, Mahidol University, Bangkok, Thailand; 2 Centre for Tropical Medicine and Global Health, Nuffield Department of Medicine, University of Oxford, Oxford, United Kingdom

## Abstract

Lorenz von Seidlein and Nicholas White introduce a Collection on Plasmodium vivax malaria.

Historically, *Plasmodium vivax* has persisted in the shadow of the more prominent *Plasmodium falciparum*. Up until 2013, *P*. *vivax* malaria was little more than a footnote in WHO reports, with *P*. *falciparum* and *P*. *vivax* malaria-attributable morbidity and mortality not disaggregated by malaria species. Yet an assumption that reduction in the disease burden of *P*. *falciparum* would be mirrored by a proportionate reduction in the *P*. *vivax* burden has turned out to be misplaced: *P*. *vivax* is a stealthier and more resilient cousin of *P*. *falciparum*. In the accompanying *PLOS Medicine* Collection, we focus on *P*. *vivax* malaria and discuss recent progress in recognising and addressing the widespread and serious disease burden. Studies addressing key research questions in the field will also be published alongside the Collection.

Although malaria elimination in Africa appears to be stagnating, there is an ongoing substantial reduction in *P*. *falciparum* malaria in Asia and the Americas. This has resulted from improvements in prevention and treatment, the sterling work of health workers, billions of dollars invested by donors, and the ongoing benefits of economic development. For such efforts to continue, there is a need to show progress, notably the certification of malaria elimination from as many countries as possible. But as *P*. *falciparum* malaria recedes in these regions, the more difficult challenge of *P*. *vivax* malaria remains, responding much more slowly to the established control methods that have driven *P*. *falciparum* to near elimination in many areas.

The population at risk for *P*. *vivax* infections includes large swathes of Asia, which host some of the most densely populated regions on the planet [1]. More than a third of the world’s population are, at any one time, potentially at risk of becoming infected with *P*. *vivax*, although in general, these risks are low. For example, a large proportion of the population of India—more than 1.3 billion people in total—remains at potential risk for *P*. *vivax* malaria. Recent epidemiological studies in low *P*. *falciparum* malaria transmission settings such as the greater Mekong Subregion have revealed the large, hidden burden of *P*. *vivax* malaria [2,3]. Compared with *P*. *falciparum*, *P*. *vivax* is less likely to kill directly but, through recurrent infections, it can cause considerable morbidity including, in children, anaemia, growth retardation, and developmental delay [4]. *P*. *vivax* infections in pregnancy cause fetal loss and reduce birthweight, compromising infant survival [5,6]. The evolving understanding of the epidemiology of *P*. *vivax* is discussed in an accompanying review [7].

Compared with *P*. *falciparum*, there is an additional stage in the life-cycle of *P*. *vivax* infection. Hypnozoites, literally sleeper cells, in the liver cause relapses of malaria [8]. But there are few hypnozoites in infected livers among the billions of hepatocytes, and there are no diagnostic tools available to diagnose people carrying them. The quiescent hypnozoites are resistant to most antimalarial drugs. Despite more than seven decades of research, only a single class of antimalarials, the 8-aminoquinolines, has been identified that prevents relapses (known as the “radical cure”). Unfortunately, all members of this class of drugs, including the recently approved long-acting tafenoquine, cause haemolysis in people with glucose-6-phosphate dehydrogenase (G6PD) deficiency [9,10]. Gene frequencies for this X-linked enzymopathy range from approximately 3% to 35% in most *P*. *vivax*-endemic countries. G6PD testing has not been made widely available and so the possibility of doing harm by causing oxidant haemolysis has markedly limited implementation of radical cure [11,12]. Individual and population risks associated with primaquine radical cure have not been defined well, particularly as G6PD deficiency protects against *P*. *vivax* malaria [13], nor has the cumulative adverse impact of not providing radical cure (i.e., the burden of relapse). However, mainly because of tafenoquine, more than a decade of recent development has resulted recently in marketing of the first point-of-care biosensors, which allow a quantitative measurement of G6PD activity [14–16]. These are necessary to avoid prescription of tafenoquine to fully G6PD-deficient hemizygotes or homozygotes, and also to partially deficient female heterozygotes, and thereby to avoid protracted haemolysis. But it is far from clear whether it will be feasible to purchase, distribute, and maintain the many biosensor units and reagents needed. To implement a safe radical cure widely, it will also be necessary to train large numbers of community health workers who are usually the first and only contact of *P*. *vivax* malaria patients. Overall, radical cure is underused, and the vast majority of *P*. *vivax* malaria patients receive only treatment with a schizontocidal drug. After variable intervals, many of these patients relapse, either symptomatically or asymptomatically, and continue to transmit *P*. *vivax* infections. These relapses which maintain *P*. *vivax* infections are a major challenge to elimination. Prevention and treatment of *P*. *vivax* malaria are discussed further in a review in the Collection [17].

Development of malaria vaccines, which has absorbed a large proportion of malaria research funds in recent decades, has concentrated almost exclusively on *P*. *falciparum*, while research on the development of new hypnozoiticidal drugs has been relatively neglected. Most antimalarial drug trials have focused on *P*. *falciparum* malaria, resulting in a robust research design for the evaluation of schizontocidal drugs. The evaluation of hypnozoiticidal drugs is more complex—how should patients be followed up after receiving a candidate radical cure regimen, and for how long? There is no generally agreed methodology for drug efficacy assessments in *P*. *vivax* malaria. The current diversity of national policies and practices for radical cure is further testament to the persistent low priority given to *P*. *vivax* malaria.

But the tide is turning in favour of confronting the challenge of *P*. *vivax* malaria. There has been a recent increase in clinical and epidemiological research on *P*. *vivax*, and elimination has become a stated goal for many countries with endemic *P*. *vivax*. Notably, Sri Lanka has managed to eliminate all malarias during a viciously fought civil war [18], and China has nearly eliminated all forms of malaria. The recent willingness of large funders to invest in malaria elimination, which must include the elimination of *P*. *vivax* malaria, is closely linked with the ambition of policymakers to achieve this goal. The political will to address *P*. *vivax* malaria is helped by technical innovations. The rapid diagnostic tests to diagnose *P*. *vivax* malaria have considerably improved over the last decade. The first biosensor to diagnose G6PD deficiency has received regulatory approval, and more tools to detect and quantify G6PD deficiency are in the development pipeline. Tafenoquine is receiving regulatory approval in more malaria-endemic countries, and large donors are backing its roll out (although the dose may be suboptimal). These developments facilitate the early diagnosis and treatment of acute *P*. *vivax* malaria and the prevention of relapse—prospects for elimination of *P*. *vivax* are discussed further in an accompanying review article in the Collection [19].
